# Grid-tied Transformer-less Boost Switched Capacitor Topology (TLBSCT) for PV applications

**DOI:** 10.1371/journal.pone.0352760

**Published:** 2026-07-06

**Authors:** Sidharth Samantara, A. Muruli Krishna, Kasinath Jena, Krishna Kumar Gupta, Dhananjay Kumar, Niraj Kumar Dewangan

**Affiliations:** 1 Department of Electrical and Electronics Engineering, ARKA JAIN University, Jharkhand, India; 2 Department of Electrical and Electronics Engineering, BIET Bhadrak, Odisha, India; 3 Department of Electrical & Instrumentation Engineering, Thapar Institute of Engineering & Technology Patiala, India; 4 Department of Electrical Engineering, Government Engineering College, Siwan, India; 5 Manipal Institute of Technology, Manipal Academy of Higher Education, Manipal, India; Vellore Institute of Technology, INDIA

## Abstract

This paper proposes a new grid-tied transformer-less boost switched capacitor topology (TLBSCT) that employs three capacitors and twelve switches to generate seven levels with a gain of three times. The salient features of the TLBSCT are its boosting capacity, zero leakage current, minimum switching devices and lower voltage stress. The capacitors of the proposed TLBSCT have self-balancing characteristics. The proposed TLBSCT offers a brief discussion of the configuration, principle of working and the design of the parameter, as well as its control scheme. In addition, a comparative study of the proposal against the current transformerless inverter (TLI) shows the better performance of the proposed approach(PA). Also, the theoretical concept and viability of the suggested design have been demonstrated by simulations and experiments. This work contributes to SDG 7: Affordable and Clean Energy by improving efficient and reliable grid-connected solar power conversion systems.

## 1. Introduction

The rising demand for renewable energy sources (RES) is driven by critical global challenges such as air pollution, climate change, and the finite availability of fossil fuels. Consequently, there is a steady global increase in the integration of RES, especially photovoltaic (PV) systems, into power grids. Power converters play a crucial role in interfacing PV systems with the electrical grid. Various configurations are employed for grid integration, primarily categorized as transformer-based inverters (TBIs) and transformerless inverters (TLIs).

In order to improve operational safety and system protection, transformer-based inverter TBI designs offer galvanic isolation between the source and load. But adding transformers decreases efficiency and power density, and it also enhances the overall cost, volume and weight of the system. A lot of people have been looking at TLI topologies as a solution to these problems recently. The advantages of TLIs over conventional TBI systems for residential solar applications include a smaller footprint, cheaper production costs, a lighter structure, less leakage current, and higher conversion efficiency [1,2]. For PV installations that are connected to the grid, a new topology for single-phase TLIs is presented in [3]. The proposed design aims to reduce leakage current by maintaining a constant common-mode voltage (CMV) and addressing issues with traditional TLIs.

TLIs offer several advantages; nevertheless, the primary negative aspect is the absence of galvanic separation between the PV array and the utility grid. This direct electrical connection causes leakage currents to flow from the PV array to the ground, which may cause additional power losses, grid current distortion, electromagnetic interference, deteriorated system efficiency and potentially safety hazards [4–6]. Several MLIs have been proposed in the literature to tackle the above problems. [7] proposes a five-level SC based neutral-point clamped (NPC) inverter by adding a SC unit to the conventional three-level NPC configuration. The architecture can provide a 5-level output voltage. The output voltage magnitude is limited to half the input voltage, giving a voltage gain of only 0.5. [8] shows the 5L-SC-ANPC and this architecture demands a high number of power switches and the related gate driver circuits, which increases the total control complexity. A hybrid inverter structure based on T-type topology and cascaded SC cells is proposed in [9]. This topology connects the neutral point of the AC output with the middle of the DC bus, thereby reducing the common-mode voltage. However, the configuration still uses a lot of components and suffers from high voltage stress in the semiconductor devices. In addition, the five-level SC based ANPC design proposed in [10] provides unity voltage gain with six switches, two diodes and three capacitors. However, the demand for higher DC-link voltage and higher RMS current leads to higher conduction losses and hence lower system efficiency.

In the ANPC design, the leakage current can be greatly reduced by connecting the grid neutral line with the middle of the DC bus [10–13]. There are a few drawbacks to this method, though. For one, the DC-link voltage must be twice the peak grid voltage. Secondly, there must be more switching components. Lastly, the leakage current problems are not entirely fixed. As a typical topology design for effectively mitigating leakage current [14–17] introduces the idea of a Virtual DC bus. By utilising a virtual DC rail, this connection permits the generation of negative voltage. Still, the complexity and cost of the aforementioned topologies skyrocket since they call for more active and passive switching components. Reference [18] proposes a seven-level switched-capacitor boost inverter, which links an output converter and a DC-link converter through a bidirectional switch to achieve seven voltage levels with a gain of just 1.5. The maximum voltage delivered to all power switches is not higher than the input DC voltage. The seven-level MLI presented in [19] produces a voltage gain of just 1.5 with nine switches and three capacitors. In reference [20], the ANPC inverter topology with seven levels, eight switches, two diodes and four capacitors is recommended. The architecture may generate multi-voltage levels, but an additional capacitor voltage balancing circuit based on closed-loop control is required to enhance the multi- voltage levels, which will raise the complexity and implementation difficulty of the whole system. The MLIs in [21] and [22] are using ten switches, two diodes and three capacitors, but the voltage gain is just 1.5. Moreover, these arrangements have a high total standing voltage per unit (TSVpu), requiring the semiconductor devices to have higher voltage ratings. According to [23,24], 7-level SC topologies include more switching components and TSV. The topologies of high-gain SCs are covered in [25,26]; the MBV of these topologies is equal to the load voltage. SC topologies with a low device count are addressed in [27,28]. However, in these topologies, the MBV is exactly the same as the load voltage. Additionally, the control techniques in [29–31] employ fuzzy logic control for enhanced energy extraction and minimized mechanical stress on the system with ultra-capacitor-based energy storage to compensate for abrupt power fluctuations and to ensure power balance during transient operating conditions. Conventional common-ground voltage-boosting topologies reported in [32–34] effectively reduce leakage current. However, these configurations require a larger number of components, resulting in increased circuit complexity and cost.

Drawing from the discussion in the preceding sections, this work introduces a TLBSCT that offers several notable advantages:

1. The PT provides a voltage gain of 3 times while utilising 12 switches.
2. The capacitors possess inherent self-balancing properties, eliminating the need for additional circuits or sensors to maintain voltage balance.
3. It uses a reduced number of switching components.
4. The switches are subjected to stresses with low voltages.
5. The PT has zero leakage current
6. Lower MBV
7. It is suitable for low-voltage applications
8. It can operate for different load power factors

The TLI structural details, working mechanism, and control strategy are thoroughly explained. The losses associated with the SC topology are also analyzed. The proposed TLI is compared with other existing designs, with a focus on its superior performance. Experimental results for both grid-connected and standalone modes are included to confirm the practicality and effectiveness of the proposed TLI.

## 2. Proposed topology

(a) **Description of the circuit diagram**

**Fig 1:**
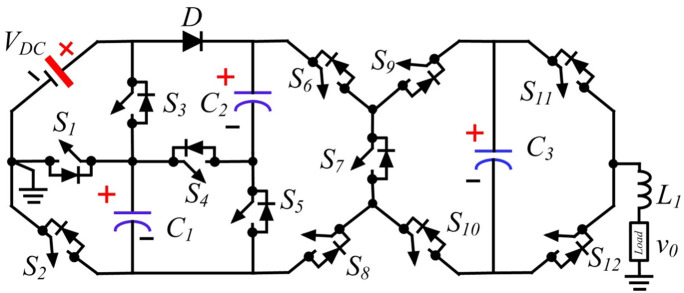
Proposed TLBSCT.

The conceptual circuit of the proposed 7-level boost SCMLI is represented in Fig 1. The PT used twelve switches, three capacitors, and a single DC supply (of $V_{DC}$ voltage) through which the output voltage can yield seven different values that include levels $\pm {\text{3}V}_{DC},\pm {\text{2}V}_{DC},\pm {\text{1}V}_{DC}$, and 0, resulting in a three times voltage gain, thus the input voltage can be attained from a lower DC voltage, such as that supplied by a PV panel. The DC supply charges the capacitors fully and there are no additional sensors needed to maintain the source voltage ($V_{DC}$) across SC in this topology, thereby reducing cost. Moreover, this topology is configured so that all power devices have lower blocking voltages as well and there is a three-voltage-boosting factor. It can push low DC voltage from PV panels, fuel cells, or electric vehicle battery storage banks up to quality AC output voltage. Table 1 illustrates the switching logic of the PT.

**Table 1:** Switching combinations.

M	Switches												E			${𝐯}_{0}$
	*S*_*1*_	*S*_*2*_	*S*_*3*_	*S*_*4*_	*S*_*5*_	*S*_*6*_	*S*_*7*_	*S*_*8*_	*S*_*9*_	*S*_*10*_	*S*_*11*_	*S*_*12*_	*C1*	*C2*	*C3*	
$\gamma_{\text{1}}$	–	1	1	–	1	–	–	1	–	1	–	1	↑	↑	–	0
$\gamma_{\text{2}}$	–	1	1	–	1	1	–	–	1	0	1	–	↑	↑	–	${\text{1}V}_{DC}$
$\gamma_{\text{3}}$	–	1	1	1	–	1	–	1	1	1	1	–	↑	↓	↑	${\text{2}V}_{DC}$
$\gamma_{\text{4}}$	–	1	1	1	–	1	1	–	–	1	1	–	↑	↓	↓	${\text{3}V}_{DC}$
$\gamma_{\text{5}}$	1	–	–	–	–	–	–	1	–	1	–	1	↓	–	–	${-\text{1}V}_{DC}$
$\gamma_{\text{6}}$	–	1	1	–	1	–	1	1	–	–	–	1	↑	↑	↓	${-\text{2}V}_{DC}$
$\gamma_{\text{7}}$	1	–	–	–	–	–	1	1	1	–	–	1	↓	–	↓	${-\text{3}V}_{DC}$

*M = Mode, E = Effect of capacitor ↓=Discharge,↑=Charging,”1”=On, “-” = no effect or off state.*

(b) **Working Principle**

The working principle of the PT is explained through seven separate operating modes

*Mode* $\gamma_{\text{1}}$. $v_{\text{0}}=\text{0}$

**Fig 2:**
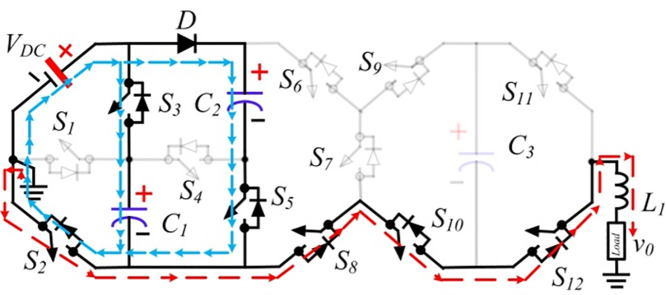
$v_{0}=0$.

In Mode $\gamma_{\text{1}}$, the output voltage is zero, as indicated by the conduction path for the load current (represented in red). The switches *S*_*2*_*, S*_*3*_*, S*_*5*_*, S*_*8*_*, S*_*10*_-, and *S*_*12*_ are in the ON state. Moreover, the capacitors C_1_, and C_2_ are both charged to $V_{DC}$, because they are both connected in parallel with the input DC source, as seen in the blue conduction line. The circuit representation corresponding to this mode is shown in Fig 2.

*Mode* $\gamma_{\text{2}}$. $v_{\text{0}}=\text{1}V_{DC}$

**Fig 3:**
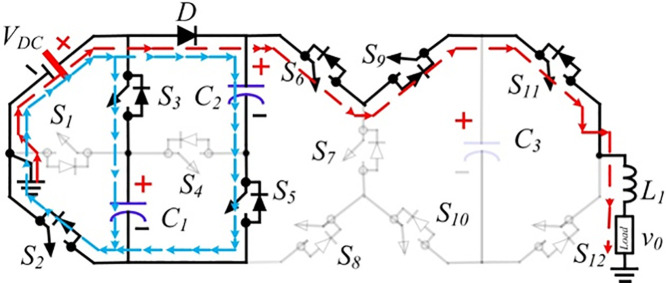
${𝐯}_{0}=1V_{DC}$.

Switches *S*_*2*_, *S*_*3*_, *S*_*5*_, *S*_*6*_, *S*_*9*_, and *S*_*11*_ are turned on, enabling the load current to follow the red-marked path, while the capacitor current flows along the blue-marked route. The output voltage is ${\text{1}V}_{DC}$, with both capacitors, C_1_ and C_2_, connected across the input $V_{DC}$ to maintain self-voltage balance. The circuit representation corresponding to this mode is illustrated in Fig 3.

*Mode* $\gamma_{\text{3}}$. $v_{\text{0}}={\text{2}V}_{DC}$

**Fig 4:**
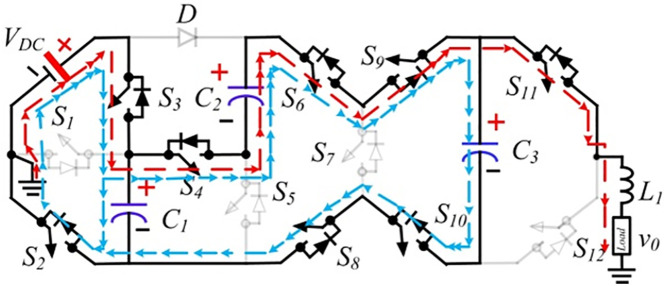
$v_{0}=2V_{DC}$.

Mode $\gamma_{\text{3}}$ is depicted in Fig 4. Switches *S*_*2*_, *S*_*4*_, *S*_*6*_, *S*_*9*_, and *S*_*11*_ are activated, allowing the load current to follow the red-marked path, while the capacitors’ current flows along the red-marked route. Capacitor C_2_ discharges through the load, increasing the output voltage to ${\text{2}V}_{DC}$ without the need for any additional switch. Meanwhile, capacitor C_1_ remains connected in parallel with the input DC supply through *S*_*2*_*,* ensuring it charges to a voltage of $V_{DC}$. C_3_ remains connected in parallel with the input DC supply and capacitor C_2_ through *S*_*8*_ and *S*_*10*_, ensuring it charges to a voltage of ${\text{2}V}_{DC}$.

*Mode* $\gamma_{\text{4}}$. $v_{\text{0}}={\text{3}V}_{DC}$

**Fig 5:**
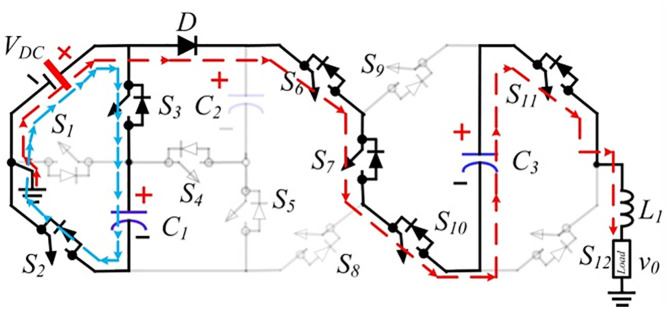
$v_{0}=3V_{DC}$.

Mode $\gamma_{\text{4}}$ is depicted in Fig 5. Switches *S*_*3*_, *S*_*4*_, *S*_*6*_, *S*_*7*_, *S*_*10*_, and *S*_*11*_ are turned on, enabling the load current to follow the red-marked path, while the capacitors’ current flows along the blue-marked path. The output voltage is ${\text{3}V}_{DC}$, with a capacitor, C_1_ connected across the input $V_{DC}$(through *S*_*3*_)to maintain self-voltage balance.

*Mode* $\gamma_{\text{5}}$. $v_{\text{0}}={-\text{1}V}_{DC}$

**Fig 6:**
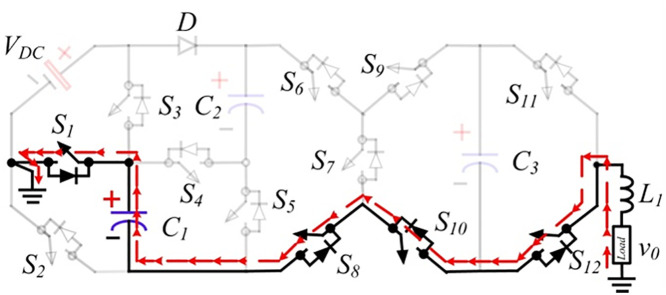
$v_{0}=-1V_{DC}$.

Mode $\gamma_{\text{5}}$ is depicted in Fig 6. During the negative half-cycle, switches *S*_*1*_, *S*_*8*_, *S*_*10*_, and *S*_*12*_ are turned ON, directing the load current along the red-marked conduction path and generating a voltage of ${-\text{1}V}_{DC}$ at the load terminals. Capacitor C_1_ discharges through the load, increasing the output voltage to ${-\text{1}V}_{DC}$.

**Fig 7:**
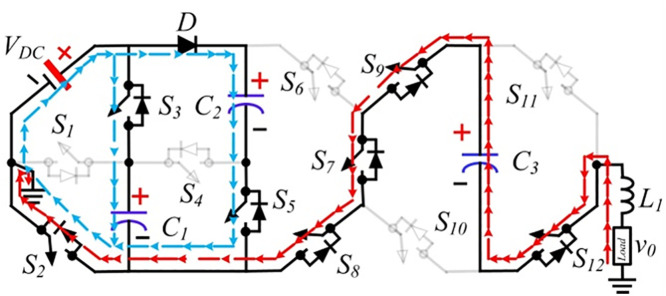
${𝐯}_{0}=-2V_{DC}(g)v_{0}=-3V_{DC}$.

Mode $\gamma_{\text{6}}$ is depicted in Fig 7. Switches *S*_*2*_, *S*_*8*_, *S*_*7*_, *S*_*6*_, *S*_*9*_, and *S*_*12*_ are turned ON, causing capacitor C_3_ to discharge during the negative cycle, generating an output voltage of ${-\text{2}V}_{DC}$. The load current follows the conduction path indicated in the red line. Simultaneously, the DC supply is connected to capacitors C_1_ and C_2_ (blue highlighted circuit), charging it (via *S*_*3*_, *S*_*5*_) $V_{DC}$.

*Mode* $\gamma_{\text{7}}$. $v_{\text{0}}={-\text{3}V}_{DC}$

**Fig 8:**
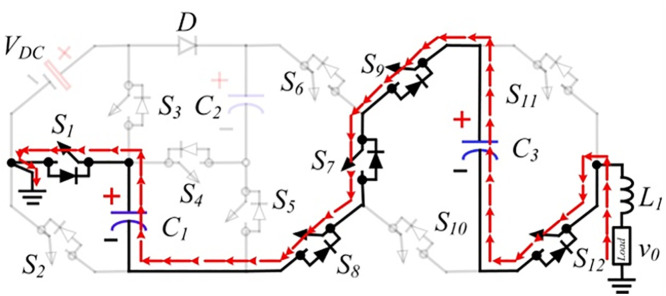
${𝐯}_{0}=-3V_{DC}$ *Mode* $\gamma_{6}$.${𝐯}_{0}={-2V}_{DC}$.

Mode $\gamma_{\text{7}}$ is depicted in Fig 8. Switches *S*_*1*_*, S*_*8*_*, S*_*7*_*, S*_*9*_*,* and *S*_*12*_ are turned ON, causing capacitors C_3_ and C_1_ to discharge during the negative cycle, generating an output voltage of ${-\text{3}V}_{DC}$. The load current follows the conduction path indicated in red.

(c) **Voltage Balancing mechanism of capacitors**

During one fundamental cycle of the load voltage, the capacitor $C_{\text{1}}$ charges to $V_{DC}$ at voltage levels of 0, ${\text{1}V}_{DC}$,${\text{3}V}_{DC}$, $\pm {\text{2}V}_{DC}$, while it discharges at${-\text{1}V}_{DC}$, ${-\text{3}V}_{DC}$. Similarly, a capacitor $C_{\text{2}}$ charges to ${\text{1}V}_{DC}$ at voltage levels of 0,$V_{DC}$,${-\text{2}V}_{DC}$, while it discharges at ${+\text{2}V}_{DC}$. Capacitor ${\text{C}}_{\text{3}}$ charges at ${+\text{2}V}_{DC}$ and discharges at ${\pm \text{3}V}_{DC}$. This continuous charging and discharging process occurs over one fundamental cycle. The series/parallel approach described in [23] can be used to charge and discharge the capacitors in the PT. Figs 2–8 depict how the capacitors charge and discharge to generate the various voltage levels and their switching pattern is summarized in Table 1. This ensures automatic self-balancing of the capacitors, stabilizing $C_{\text{1}}$ and $C_{\text{2}}$*,* and $C_{\text{3}}$at ${\text{2}V}_{DC}$.

(d) **Guidelines for the Selection of the rating of a capacitor**

This subsection represents a critical aspect of the proposed SC-based inverter topology. Numerous factors, including the type of connected load, peak load current ($I_{max}$), and maximum discharge duration $(\theta_{x}-\theta_{y}$), influence the choice of capacitor ratings. In light of this, the charge${\Delta Q}_{i}$ released by the $i^{th}$ capacitor during operation can be mathematically expressed as [5]:


$$\begin{array}{c} {\Delta Q}_{i}=\int_{\theta_{x}}^{\theta_{y}}I_{max}\text{sin}(\omega \theta -\emptyset )d\theta \end{array}$$ (1)

Consider the worst-case scenario, (i.e., for pure resistive load [23]), maximum discharge can be expressed as:


$$\begin{array}{c} {\Delta Q}_{i}=C\times {\Delta V}_{Ci} \end{array}$$ (2)

$$\begin{array}{c} {\Delta V}_{C\text{1}}=\frac{\text{1}}{\omega C\text{1}}\int_{\theta_{\text{1}}}^{{\pi -\theta }_{\text{1}}}\frac{\text{1}.\text{5}V_{DC}}{R}d\theta \end{array}$$ (3)

$$\begin{array}{c} {\Delta V}_{C\text{1}}=\frac{{\text{1}.\text{5}V}_{DC}}{{\omega RC}_{\text{1}}}\left[\pi -\text{2}\theta_{\text{1}}\right] \end{array}$$ (4)

$$\begin{array}{c} {\Delta V}_{C\text{2}}=\frac{\text{1}}{\omega C\text{2}}\int_{\theta_{\text{2}}}^{{\pi -\theta }_{\text{2}}}\frac{\text{2}V_{DC}}{R}d\theta \end{array}$$ (5)

$$\begin{array}{c} {\Delta V}_{C\text{2}}=\frac{{\text{2}V}_{DC}}{{\omega RC}_{\text{2}}}\left[\pi -\text{2}\theta_{\text{2}}\right] \end{array}$$ (6)

$$\begin{array}{c} {\Delta V}_{C\text{3}}=\frac{\text{1}}{\omega C\text{3}}[\int_{\theta_{\text{1}}}^{{\pi -\theta }_{\text{1}}}\frac{\text{1}.\text{5}V_{DC}}{R}d\theta +\int_{\theta_{\text{2}}}^{{\pi -\theta }_{\text{2}}}\frac{\text{2}V_{DC}}{R}d\theta ] \end{array}$$ (7)

$$\begin{array}{c} {\Delta V}_{C\text{3}}=\frac{V_{DC}}{{\omega RC}_{\text{3}}}\left[\text{3}.\text{5}\pi -\text{4}\theta_{\text{2}}-\text{3}\theta_{\text{1}}\right] \end{array}$$ (8)

$$\begin{array}{c} \theta_{\text{1}}={\text{sin}}^{-\text{1}}\left(\frac{\frac{\text{1}}{\text{2}M}}{\text{2}\pi f}\right) \end{array}$$ (9)

$$\begin{array}{c} \theta_{\text{2}}={\text{sin}}^{-\text{1}}\left(\frac{\frac{\text{1}.\text{5}}{\text{2}M}}{\text{2}\pi f}\right) \end{array}$$ (10)

Hence, the required capacitance values are obtained as:


$$\begin{array}{c} C_{optimum,i}>\frac{{\Delta Q}_{Ci}}{{\Delta V}_{Ci}} \end{array}$$ (11)

Where,${\Delta Q}_{Ci}$ represents the charge variation of capacitor ($C_{i}$) during the charging/discharging interval, and ${\Delta V}_{Ci}$ denotes the allowable voltage ripple across capacitor ($C_{i}$).

(e) **Analysis of leakage current**

**Fig 9:**
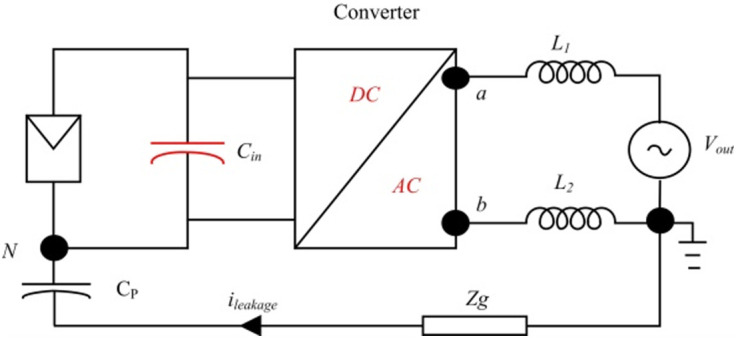
Typical TLI.


Fig 9 illustrates a typical TLI configuration [14]. The overall CMV is defined by [16] as:

$$\begin{array}{c} U_{TCM}=\frac{\left(v_{aN}+v_{bN}\right)}{\text{2}}+\left(v_{aN}-v_{bN}\right)\left(\frac{L_{\text{2}}-L_{\text{1}}}{\text{2}\left(L_{\text{1}}+L_{\text{2}}\right)}\right) \end{array}$$ (12)

Where $v_{aN}$: and $v_{bN}$ denotes the voltage difference between node ‘a’ and ‘b’ w.r.t PV module’s neutral point ‘N’. And $C_{P}$: parasitic capacitance of the PV module.

The leakage current $\left(i_{lc}\right)$ can be illustrated as


$$\begin{array}{c} i_{lc}=C_{P}\frac{{dU}_{TCM}}{dt} \end{array}$$ (13)

In this TLBSCT, $L_{\text{2}}=\text{0}$,$v_{bN}=\text{0}$, $v_{aN}=V_{DC}$. So $U_{TCM}=\text{0}$ therefore, $i_{lc}$ is zero.

## 3. Modulation pattern

**Fig 10:**
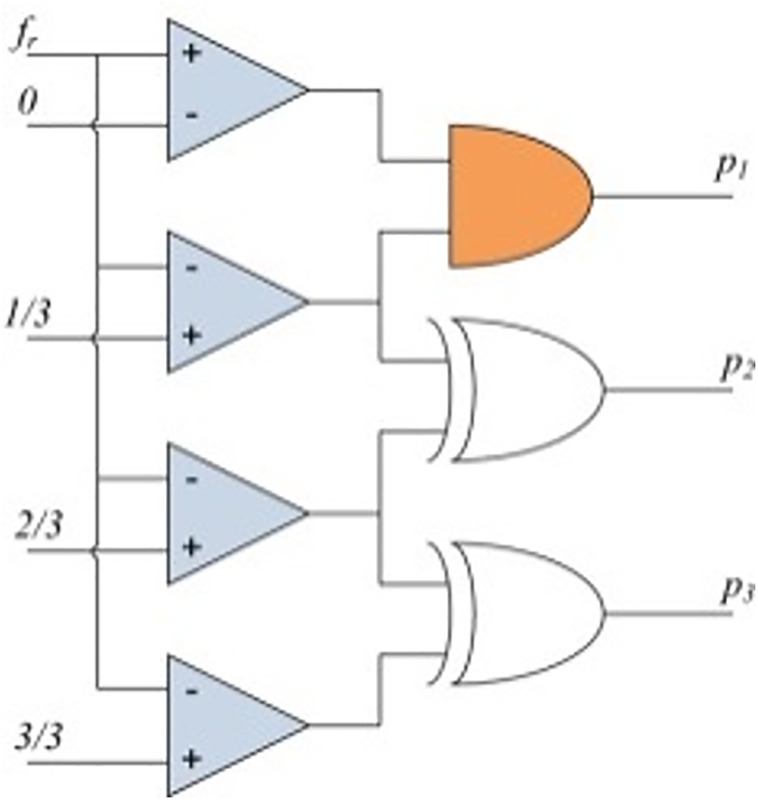
Modulation scheme for positive level.

**Fig 11:**
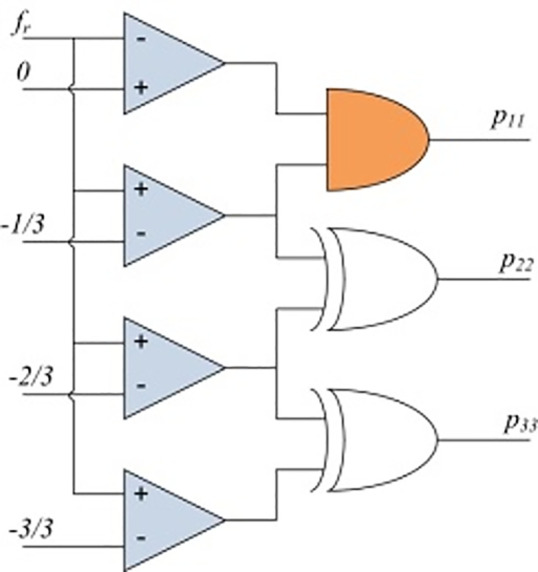
Modulation scheme for positive level.

**Fig 12:**
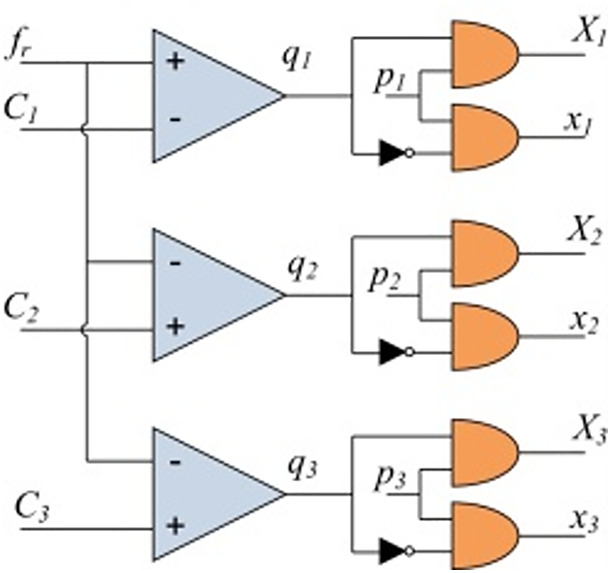
Modulation scheme for Negative level.

**Fig 13:**
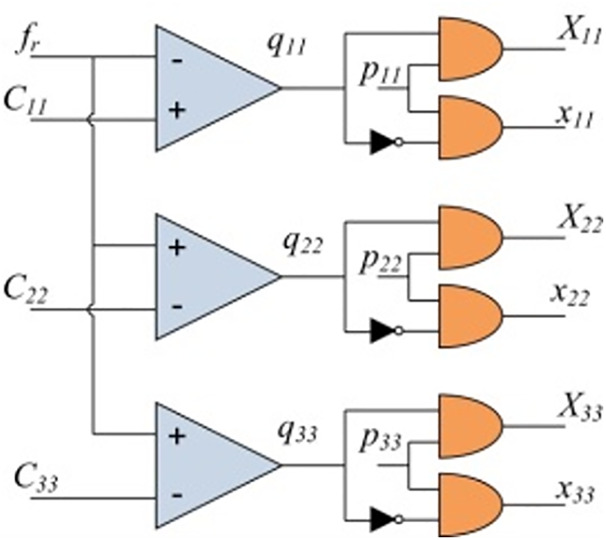
Modulation scheme for Negative level.

**Fig 14:**
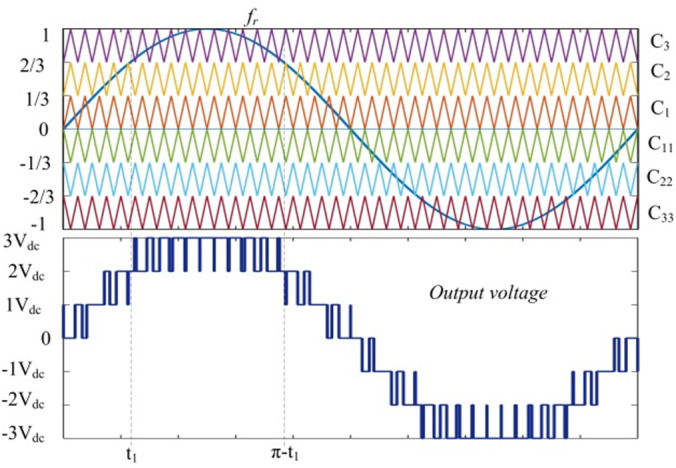
Carrier, reference and output voltage.

To ensure smooth switching operation in MLIs, various modulation strategies have been developed, including both low and high switching frequency PWM techniques. Figs 10–13 display the switching logic for the PT. In this control strategy, the modulating signal (*fr*) is initially compared with eight fixed reference levels (${\text{0}}^{+}$,1/3, 2/3, 1,${\text{0}}^{-}$,-1/3, −2/3, −1). The resulting comparator outputs are processed through AND and EX-OR logic gates to generate the signals (P_1_, P_2_, P_3_) for the positive half cycle and (P_11_, P_22_, P_33_) for the negative half cycle, as illustrated in Fig 10 and Fig 11. Subsequently, the same modulating signal (*fr*) is compared with six high-frequency carrier signals, namely *C*_*1*_*, C*_*2*_*, and C*_*3*_ for the positive half cycle and *C*_*11*_*, C*_*22*_*, and C*_*33*_ for the negative half cycle, as depicted in Fig 12 and Fig 13. This comparison produces the signals *q*_*1*_*, q*_*2*_*, q*_*3*_*, q*_*11*_*, q*_*22*_*,* and *q*_*33*_. These signals, together with their complementary versions, are then applied to separate AND gates along with (*P*_*1*_*, P*_*2*_*, P*_*3*_) and (*P*_*11*_*, P*_*22*_*, P*_*33*_). As a result, the intermediate gating signals *X*_*1*_*, x*_*1*_*, X*_*2,*_ *x*_*2*_*, X*_*3*_*, x*_*3,*_ *X*_*11*_*, x*_*11*_*, X*_*22,*_ *x*_*22*_*, X*_*33*_ and *x*_*33*_ are obtained. Finally, all of these signals are combined using OR gates to generate the final gate pulses required for operating switches (*S*_*1*_) to (*S*_*12*_). Fig 14 depicts output voltage levels obtained by the implemented modulation scheme.

## 4. Losses analysis

**Fig 15:**
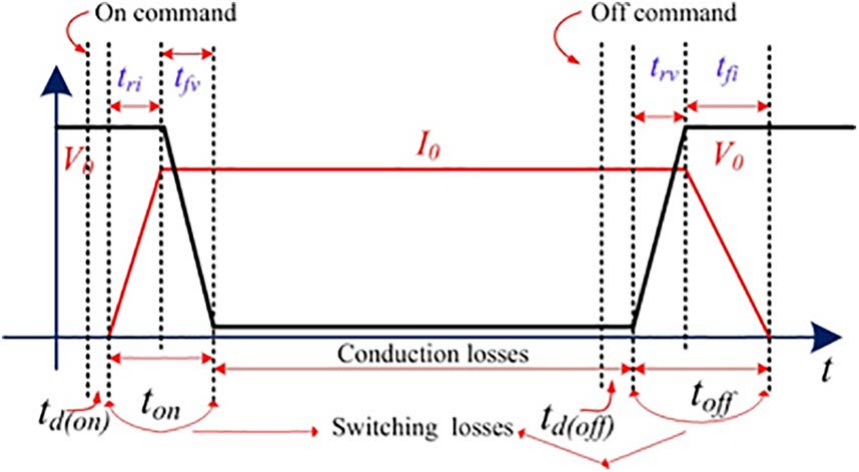
Voltage and current across the transistor during switching with overlapping.

A SCMLI experiences the following losses, namely: conduction and switching and capacitor losses [14,16,17]. Fig 15 depicts the waveforms of voltage and current during switching with overlapping characteristics.

(a) **Power losses in capacitors**

The power losses due to capacitor ripples and equivalent series resistance (ESR) are expressed as:


$$\begin{array}{c} P_{RippleLosses}=\frac{f_{o}}{\text{2}}C{\left({\Delta V}_{c}\right)}^{\text{2}} \end{array}$$ (14)

$$\begin{array}{c} P_{ConductionlossesinC}=\frac{R_{ESR}f_{o}}{\text{2}}\int_{t_{x}}^{t_{y}}{i^{\text{2}}}_{C}dt \end{array}$$ (15)

(b) ***Switching Power Losses:*** In switching processes of a power switch, there are switching losses due to inherent delays. Therefore, the power dissipated while the device turns on and off can be expressed as follows [14]:

$$\begin{array}{c} W_{loss,sw,on}=\int_{\text{0}}^{t_{on}}vidt \end{array}$$ (16)

$$\begin{array}{c} W_{loss,sw,on}=\int_{\text{0}}^{t_{tri}}V_{\text{0}}idt+\int_{\text{0}}^{t_{fv}}I_{\text{0}}vdt \end{array}$$ (17)

$$\begin{array}{c} W_{loss,sw,on}=\int_{\text{0}}^{t_{tri}}\frac{V_{o}I_{o}}{t_{ri}}tdt+\int_{\text{0}}^{t_{fv}}\frac{V_{o}I_{o}}{t_{fv}}tdt \end{array}$$ (18)

$$\begin{array}{c} W_{loss,sw,on}=\frac{\text{1}}{\text{2}}V_{\text{0}}I_{\text{0}}\left(t_{ri}+t_{fv}\right) \end{array}$$ (19)

$$\begin{array}{c} W_{loss,sw,on}=\frac{\text{1}}{\text{2}}V_{\text{0}}I_{\text{0}}\left(t_{on}\right) \end{array}$$ (20)

$$\begin{array}{c} P_{loss,sw,on}=\frac{\text{1}}{\text{2}}V_{\text{0}}I_{\text{0}}t_{on}f_{s} \end{array}$$ (21)

Similarly, the turn-off power losses in terms of voltage and current can be expressed as


$$\begin{array}{c} P_{loss,sw,on}=\frac{\text{1}}{\text{2}}V_{\text{0}}I_{\text{0}}t_{off}f_{s} \end{array}$$ (22)

Hence, the total switching losses of a power switch can be obtained as


$$\begin{array}{c} P_{loss,sw,on}=\frac{\text{1}}{\text{2}}V_{\text{0}}I_{\text{0}}(t_{on}+t_{off})f_{s} \end{array}$$ (23)

(c) ***Conduction Power Losses:*** The conduction loss of the transistor and diode of a particular power switch are obtained as [16]:

$$\begin{array}{c} \begin{array}{c} P_{ConductionLossesofTransistor}=\\ =V_{on,sw}I_{sw,avg}+R_{on,sw}\overset{\text{2}}{\underset{sw,rms}{I}} \end{array} \end{array}$$ (24)

$$\begin{array}{c} P_{ConductionLossesofDiode}=V_{on,d}I_{d,avg}+R_{on,d}\overset{\text{2}}{\underset{d,rms}{I}} \end{array}$$ (25)

The following practical switching parameters have been used for the PT Table 2

**Table 2:** Practical switching parameters.

Parameter	Practical value
MOSFET ON resistance ($R_{on}$)	0.08–0.12 Ω
Diode forward voltage ((V_f_))	0.8–1.2 V
Capacitor ESR	0.05–0.15 Ω
Dead-time interval	1–2 µs
Gate driver delay	100–200 ns
Switching frequency	2000Hz

## 5. Result discussion

The conceptual design of the PT has been verified through MATLAB simulations and tests performed on a laboratory prototype. The parameters employed for both evaluation methods are listed in Table 3.

**Table 3:** Parameters for the validation of TLBSCT.

Parameter	Specification
MOSFET	IRFP260N — 200 V, 20 A
Load	Z_1_ = 50Ω, Z_2_ = 30Ω,
Carrier frequency	2kHz
*V*_*DC*_	50V
Capacitors (*C*_*2*_ = *C*_*1,*_ *C*_*3*_)	*C*_*1*_ = *C*_*2*_ = *1600*μF*, C*_*3*_ = *2100*μF
*f*	50Hz
Modulation index (M)	0.95

(a) **Simulation investigation**

**Fig 16:**
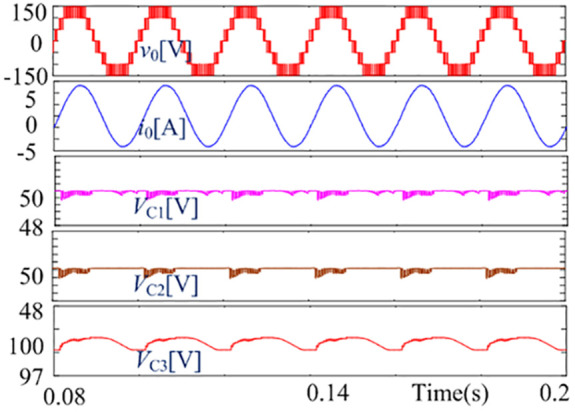
Simulation Waveforms under the steady state.

**Fig 17:**
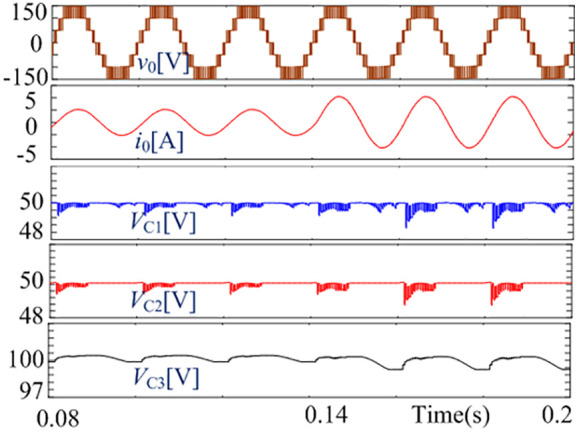
Simulation Waveforms under sudden change in load.

**Fig 18:**
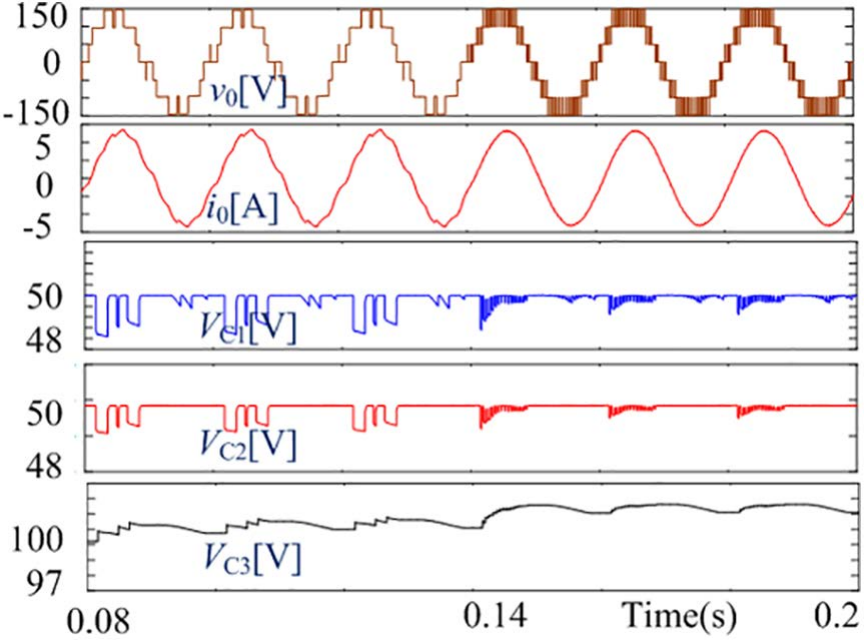
Simulation Waveforms under change in switching frequency.

Fig 16 presents the steady-state behavior of the proposed TLBSCT. It can be seen that the circuit delivers a 7-level waveform reaching a peak of 150 V, while the capacitor voltages stay automatically regulated around their intended reference values. When the PT is subjected to sudden variations in load and frequency, as illustrated in Fig 17 and Fig 18, the output voltage and the SC voltages remain stable. This confirms that the inverter maintains reliable performance under dynamic operating conditions.

**Fig 19:**
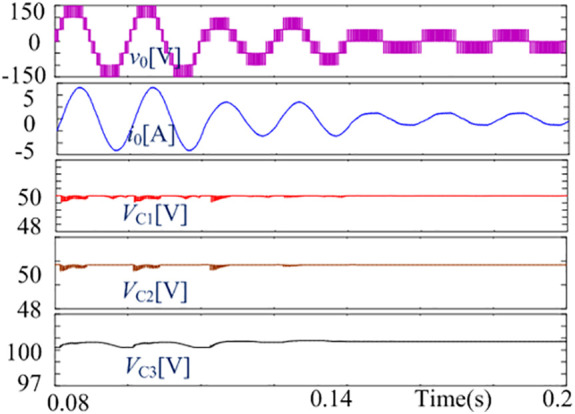
Simulation Waveforms under the change in modulation index.

**Fig 20:**
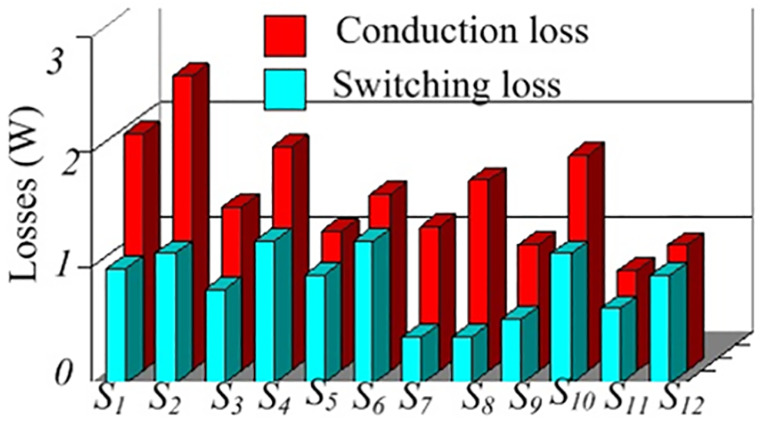
Losses distribution graph of individual switches.

**Fig 21:**
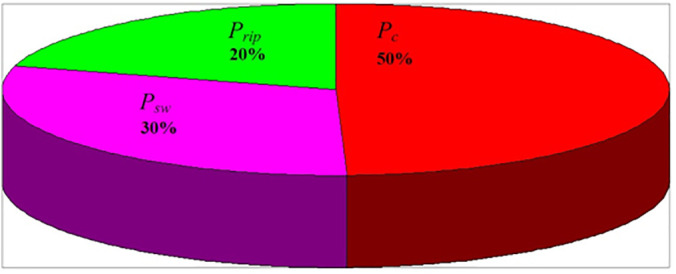
Different power losses distribution graph.

Fig 19 shows the shifts of the modulation index (M) from 0.95 to 0.75 and from 0.75 to 0.5. It is found that the decrease in the “M” results in a decrease in the magnitude of output voltage and output current, respectively. However, the PT can still maintain the voltages of SCs stable and exhibits good capacitor self-balancing performance under diverse modulation settings. Also, as shown in Fig 20 and Fig 21 are the distribution of power loss among the PT switches respectively.

(b) **Experimental study**

**Fig 22:**
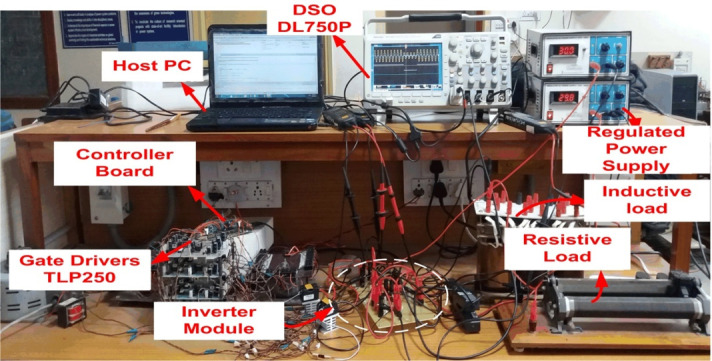
Experimental setup.

The feasibility of the PT has been demonstrated through tests carried out on a laboratory-built prototype. The module underwent testing under diverse conditions to assess the inverter’s performance. Figs 22 depict the experimental setup along with the corresponding outputs observed under different scenarios.

**Fig 23:**
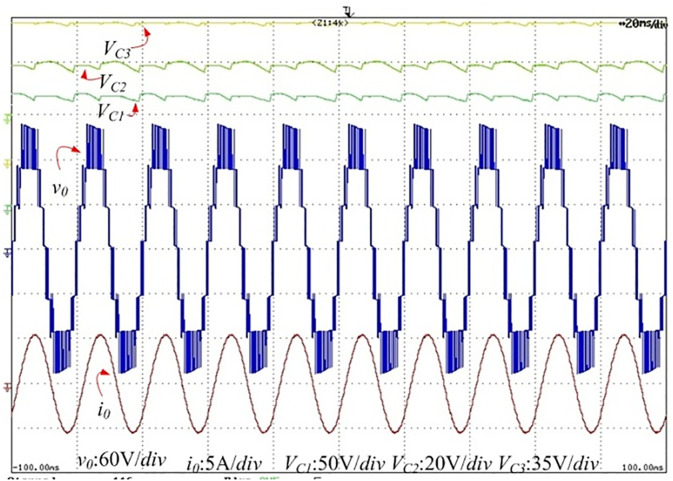
Steady state.

**Fig 24:**
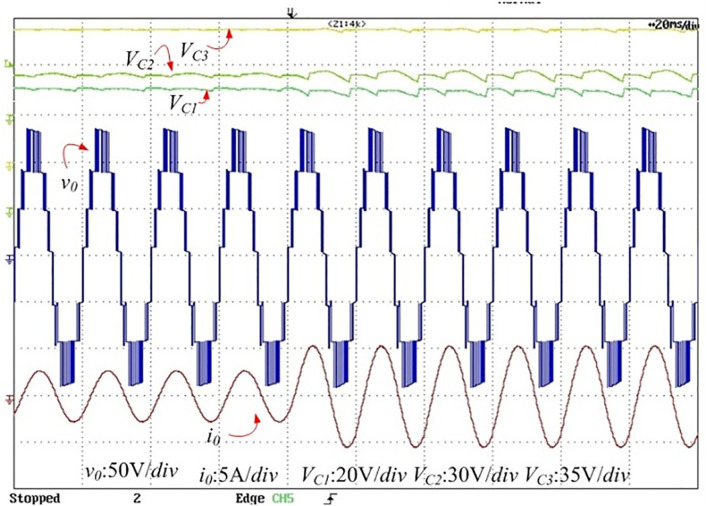
Load change.

**Fig 25:**
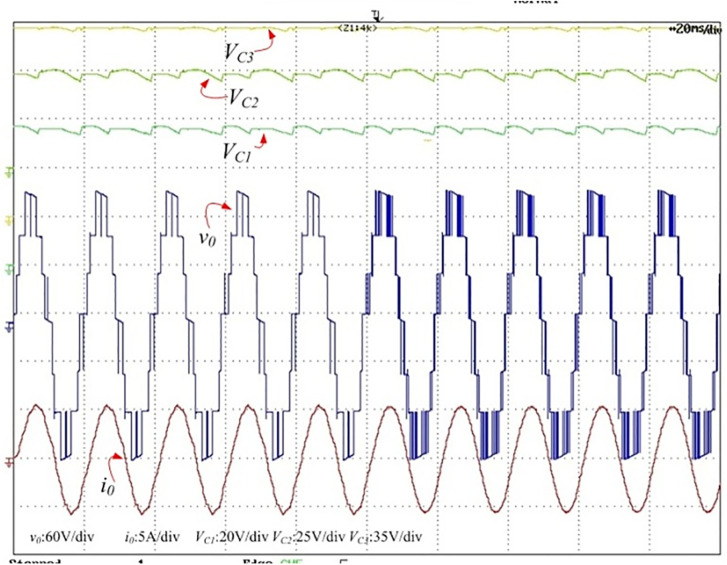
Switching frequency.

Fig 23 shows the waveforms of the R-L load in a steady-state condition. The uniform voltage across all three capacitors indicates effective voltage balancing. The waveforms for a change in RL load are shown in Fig 24. Output voltage comprises 7 levels with each level being 50V. The boost ratio is achieved to be 3, which indicates that the PT has the ability to provide an output of a maximum 150 V.It is seen from Fig 25 that the output voltage levels are consistent and not impacted by the frequency fluctuations as the switching frequency varies from 200 Hz to 2000 Hz. Moreover, the capacitor voltages are self-balanced, hence allowing stable operation for variations of switching frequency.

**Fig 26:**
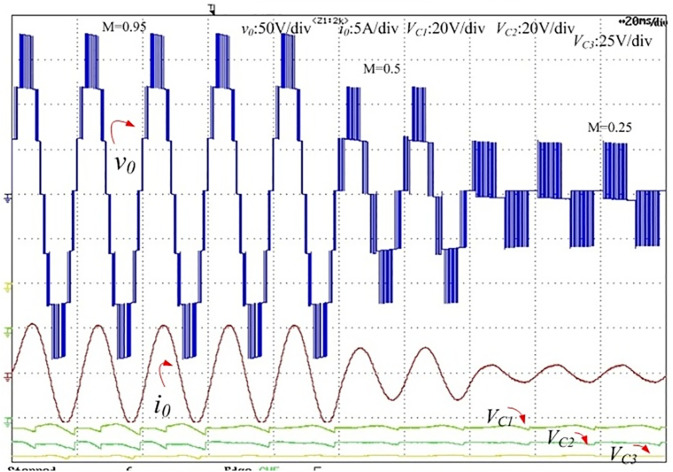
Modulation index.

**Fig 27:**
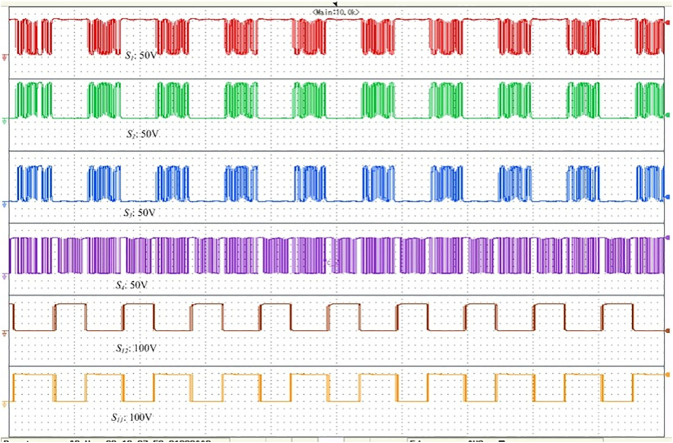
Switch voltage.

**Fig 28:**
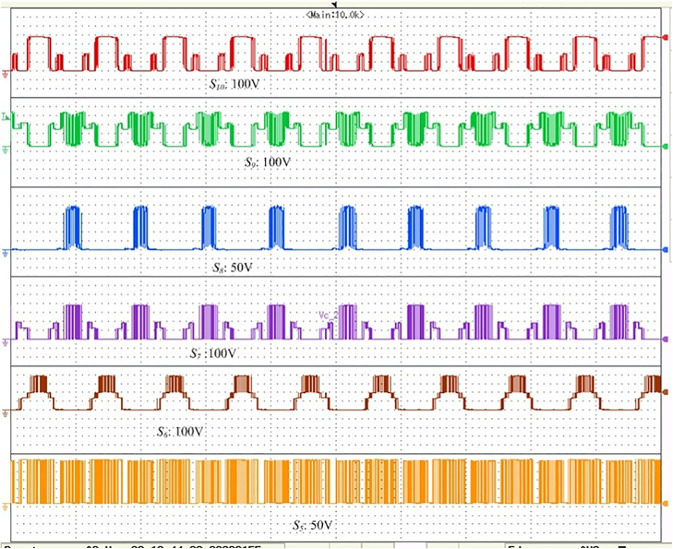
Switch voltage.

**Fig 29:**
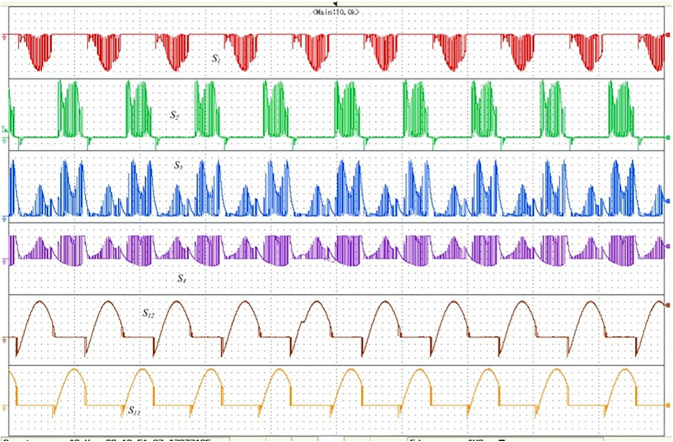
Switch current.

**Fig 30:**
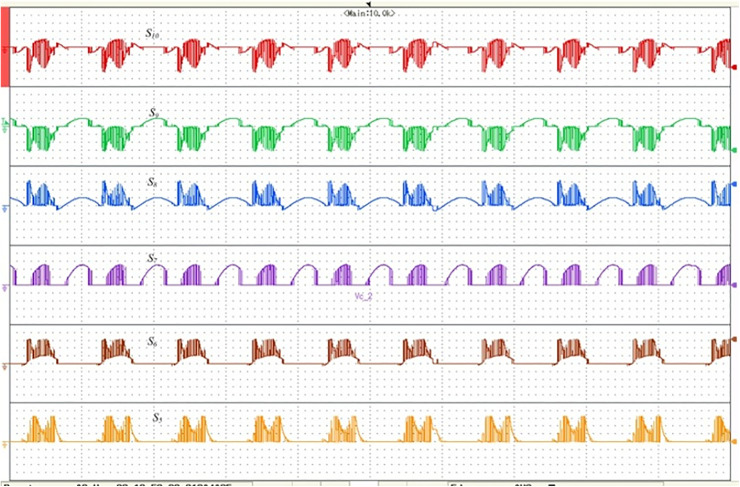
Switch current.

**Fig 31:**
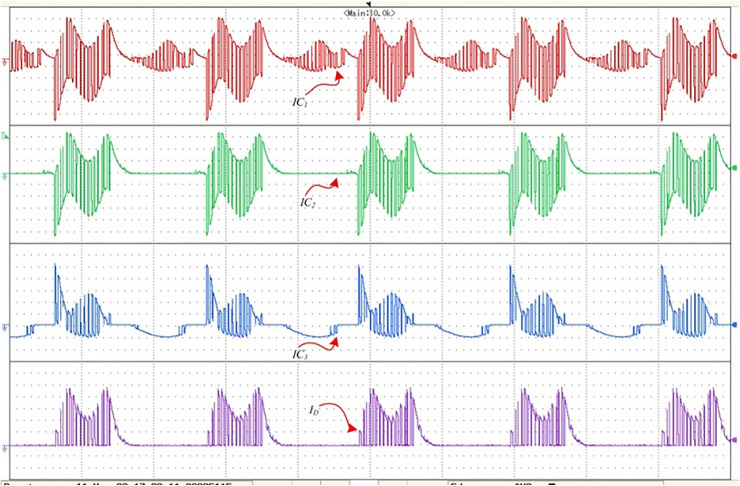
Capacitor diode voltage and current.

**Fig 32:**
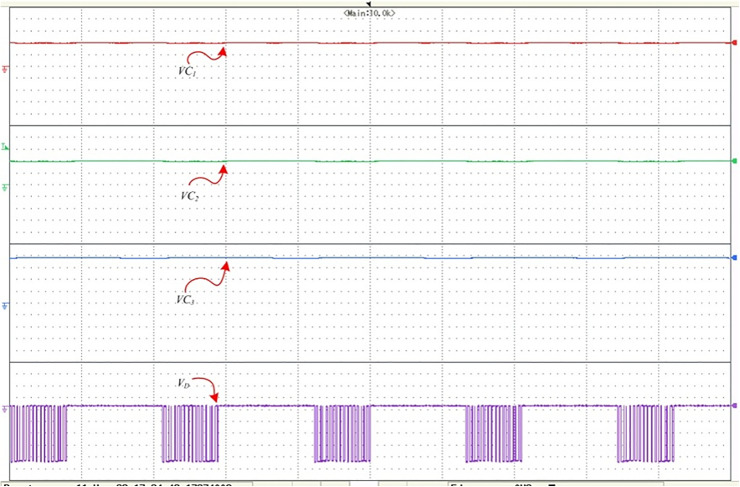
Capacitor diode voltage and current.

**Fig 33:**
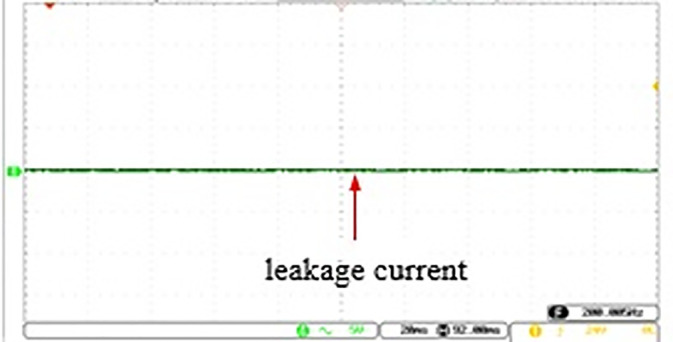
Leakage current.

**Fig 34:**
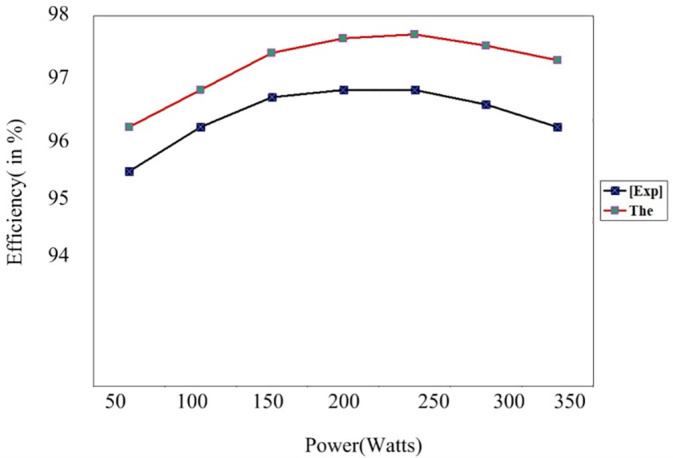
Simulation and experimental efficiency- power curve.

**Fig 35:**
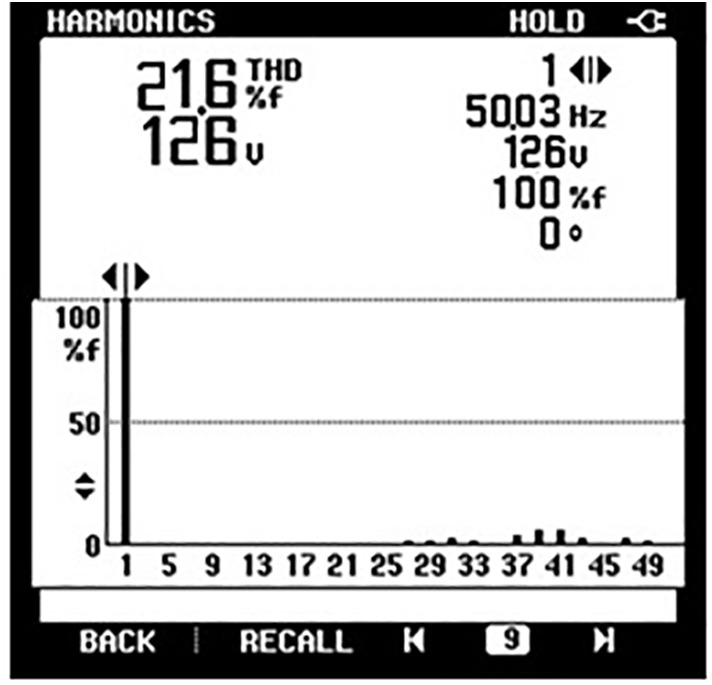
Voltage THD.

**Fig 36:**
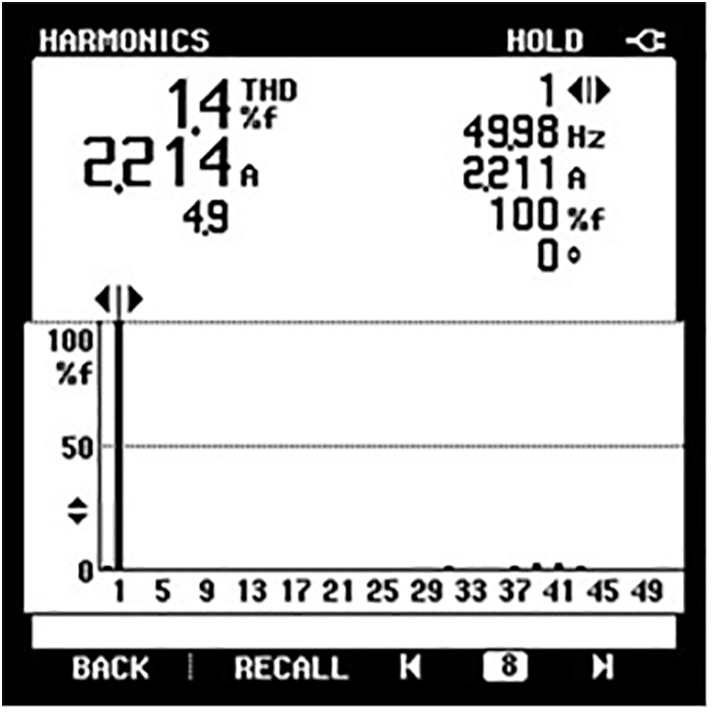
Current THD.

The inverter output is changed from 7-levels to 5- levels and subsequently to 3- levels as the M is changed (0.98 to 0.6 then to 0.3) as shown in Fig 26. This demonstrates that the PT can consistently generate a broad spectrum of voltage levels across a variety of modulation index values. Figs 27–30 show the voltage and current stresses of each switch. The voltage stress on switches is found to be below the load voltage. Figs 31 and 32 show the Capacitor, diode voltage and current. Moreover, the leakage current is observed to be zero as shown in Fig 33. The load versus efficiency curve has been plotted and the efficiency is seen to peak at about 200W in Fig 34. Voltage and current results from the THD analysis are displayed in Figs 35 and 36. The test outcomes demonstrate that the inverter performs well in steady state and shows strong dynamic behavior when responding to changes in its input conditions.

(c) **Grid-connected mode:**

When connected to the grid, this mode allows the inverter to inject a sinusoidal current that is in synchronization with the grid voltage and has minimal harmonic distortion. Using a Proportional-Resonant (PR) controller, which guarantees zero steady-state error under sinusoidal reference conditions and gives a high gain at the fundamental frequency, the PT improves power quality and achieves precise current tracking [24]. The Proportional-Integral-Derivative (PID) controller’s transfer function is given by:


$$\begin{array}{c} G_{PR}(\text{s})=K_{P}+\frac{{\text{2}K}_{r}\omega_{c}s}{S^{\text{2}}+{\text{2}\omega }_{c}s+\overset{\text{2}}{\underset{\text{0}}{\omega }}} \end{array}$$ (26)

The parameters $k_{P}$,$k_{r}$ $\omega_{c}$ denote the proportional gain, the infinite gain, and the resonant frequency, respectively. These parameters are critical for defining the behavior of the controller towards variations, the amplification at the desired frequency and the bandwidth around the resonant point.

**Fig 37:**
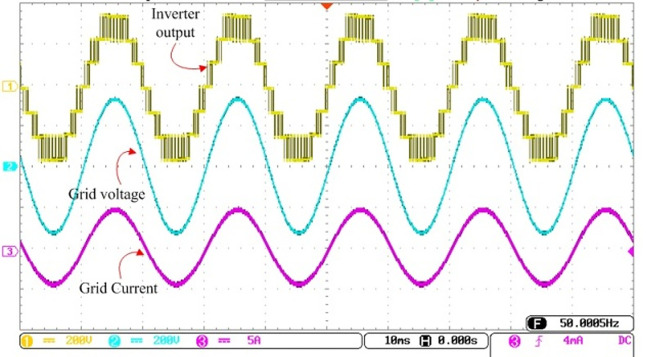
Grid Tied Results.

To confirm the appropriateness of the PR controller for grid-connected operation, the suggested system was experimentally investigated using a scaled-down prototype. The parameters used in the test setup are given in Table 4 and the corresponding experimental findings are shown in Fig 37. The inverter generates a seven-level output waveform, with a peak amplitude of 400 V. Moreover, the experimental results demonstrate that the PT provides 0.3 kW active power and the grid voltage and current are in phase. This phase alignment verifies that the inverter operates at a unity power factor. Overall, the findings confirm that the PT performs effectively and maintains dependable operation under grid-connected conditions.

**Table 4:** Grid-tied parameters.

Parameters	Value
Rated power	0.5 kW
Fillter	3 mH, 0.1Ω
RMS grid voltage	250 V
Frequency of the grid and switching	50 Hz, 2 kHz

## 6. Comparative assessment

Several inverter topologies that have been reported previously are compared with the PT in Table 5. The topologies are evaluated based on key design and performance parameters including efficiency, TSVpu, MBV, N_SW_, N_C_, N_D_, Ndri, and gain.

**Table 5:** Comparative Evaluation of Existing 7-Level TLI Configurations.

	Componets				Parameters				
**Ref.**	**A**	**B**	**C**	D	E	**F**	**G**	H	I
[35]	10	3	2	8	7.33	6	3	0	97.65
[4]	12	2	0	11	5	2	3	NR	97.7
[5]	11	3	0	10	6	3	3	0	97.4
[6]	12	4	0	12	7.5	4	4	NR	97.62
[7]	6	4	4	6	7	3	3	NR	NR
[8]	11	3	3	11	6.66	2	3	NR	96.5
[9]	16	2	0	14	5.33	1	3	NR	NR
[36]	14	2	2	14	5.33	1	3	NR	91.7
[11]	10	3	2	8	8.66	1.5	1.5	0	97.25
[12]	12	4	4	12	5.33	1	3	NR	91.1
[10]	10	3	2	9	6.67	1	1.5	NR	96.37
[14]	9	3	0	8	5.33	1	1.5	NR	NR
[15]	6	4	4	6	6.67	4	3	0	NR
[19]	16	2	0	16	5.33	1	3	NR	94
[20]	12	4	4	11	9.33	2	1.5	0	97.5
[21]	10	2	0	8	10.66	1.5	1.5	NR	97.6
[22]	7	2	2	7	7.33	1.5	1.5	NR	96
[23]	10	2	0	10	6.67	4	3	NR	96.3
[24]	12	3	2	12	7.33	3	3	0	96.95
[27]	10	2	1	10	6.3	3	3	NR	NR
[28]	7	3	2	7	7	3	3	NR	NR
[PT]	12	3	1	12	6.66	2	3	0	97.84

A:no.of switches(N_SW_), B:no. of capacitors (N_C_), C:no.of diodes (N_D_), D: no. of drivers (N_dri_), E: TSVpu, F: MBV, G: Gain, H: leakage current, I:% of Efficiency, NR: not reported

The PT uses fewer switches than the topologies described in [9,36], and [19], which each have 14–16 switches. Despite the fact that some designs, like [7] and [15], employ just 6 switches, and [22] and [28] use 7 switches, these designs sacrifice other crucial features like efficiency, diode count, or voltage stress. The PT outperforms the competition while keeping the number of switches balanced, as shown in [4,6,12,20], and [24].

In comparison to topologies [6,7,12,15], and [20], which have 4 capacitors, the PT employs just 3 capacitors. The circuit complexity, cost, and capacitor voltage balance difficulties are all reduced as a result of the lower capacitor count. Minimal capacitor usage is achieved in a few of the topologies, including [4,9,36,21–23], and [27], but these topologies typically display reduced efficiency or increased voltage stress.

The PT uses just one diode, which is much less than the four diodes needed in [7,12,15], and [20], as well as the two diodes used in [35,11,10,22,24], and [28]. Conduction losses are reduced and the converter structure is simplified by using a lesser number of diodes.

With 12 driver circuits, the PT is similar to topologies [6,12], and [24] and has the same number of switches. Some topologies, such those in [35,11,22], and [28], have lower gain or higher voltage stress, but fewer drivers are needed for them. The increased voltage boosting capability and greater efficiency of the driver in the PT make it justified.

Several existing topologies have lower TSVpu values than the PT (6.66 vs. 7.33, 7.50, 8.66, 9.33, 10.66, and 7.30, respectively) [35,6,7,11,20,21,28] (7). Despite having somewhat lower TSVpu values, topologies like [4,9,36,12,14], and [19] either have a higher number of switches needed or are less efficient.

In comparison to references [35,6,7,15], and [23], where the maximum blocking voltage (MBV) falls between 3 and 6, the PT’s MBV of 2 is lower. Reliability is improved as a result of reduced voltage stress on switches. Although some topologies manage to get an MBV of 1, these designs necessitate additional switches or offer reduced efficiency and gain (for example, [9,11,12,36,10], and [14]).

Like most reported topologies, the PT yields a voltage gain of 3. This includes topologies [4,5,35,7–9,36,12,15,23,24,27], and [28]. In addition, it achieves better results than topologies [11,10,14,20,21], and [22], which provide a lesser increase of only 1.5. A larger gain of 4 is achieved by topology [6], but it comes at the cost of more capacitors and higher TSVpu and MBV.

The removal of leakage current is another significant benefit of the PT. In keeping with previous research [35,5,11,15,20,24], the PT is well-suited for safe and high-performance power conversion applications due to its nearly negligible leakage current. On the other hand, leakage current performance is not reported by several topologies, including [4,6–9,36,12,10,14,19,21–23,27], and [28].

When compared to all other documented topologies, this one outperforms them all (97.65%, (5) 97.7%, (7) 97.62%), (22) 97.5%, (23) 97.6%). Reduced power losses and improved overall energy conversion capabilities are demonstrated by the greater efficiency of the suggested design.

## 7. Conclusion

A new seven-level three- TLBSCT for the grid-connected photovoltaic application is proposed in this study. The PT possesses many desirable advantages such as zero leakage current, decreased component count, lower voltage stress across semiconductor devices, voltage boosting capability and intrinsic capacitor self-balancing operation. The operating modes, the parameter selection and the control approach of the PT are examined in detail. Furthermore, a comprehensive comparison with recently proposed TLIs demonstrates that the proposed design is better in terms of performance and efficiency. The simulation and experimental studies under various loading circumstances show the effectiveness and practical viability of the TLBSCT. The benefits of the PT are suitable for solar PV coupled to grid applications.
